# Vaccine Targeting Alpha 1D-Adrenergic Receptor Improved Metabolic Syndrome in Mice

**DOI:** 10.1007/s10557-022-07418-9

**Published:** 2023-01-19

**Authors:** Xin Li, Wenrui Ma, Yanzhao Zhou, Chang Li, Dingyang Shi, Wenlong Kuang, Jiacheng Wu, Yuhua Liao, Zhihua Qiu, Zihua Zhou

**Affiliations:** 1 Department of Cardiology, Union Hospital, Tongji Medical College, Huazhong University of Science and Technology, Wuhan, 430022 China; 2 Hubei Key Laboratory of Biological Targeted Therapy, Union Hospital, Tongji Medical College, Huazhong University of Science and Technology, Wuhan, 430022 China; 3 Hubei Provincial Engineering Research Center of Immunological Diagnosis and Therapy for Cardiovascular Diseases, Union Hospital, Tongji Medical College, Huazhong University of Science and Technology, Wuhan, 430022 China; 4 Department of Cardiology, Institute of Cardiology, Hubei Key Laboratory of Biological Targeted Therapy, Union Hospital, Tongji Medical College, Huazhong University of Science and Technology, No. 1277 Jiefang Avenue, Wuhan, 430022 China

**Keywords:** α1-adrenergic receptor, immunotherapy, metabolic syndrome, sympathetic nervous system, vaccine

## Abstract

**Purpose:**

Metabolic syndrome (MetS) is a complex chronic disease that includes obesity and hypertension, with rising evidence demonstrating that sympathetic nervous system (SNS) activation plays a key role. Our team designed a therapeutic vaccine called ADRQβ-004 targeting the α1D-adrenergic receptor (α1D-AR). This study was performed to investigate whether the ADRQβ-004 vaccine improves MetS by modulating SNS activity.

**Methods:**

C57BL/6N mice were fed a high-fat diet (HFD) and N^ω^-nitro-L-arginine methyl ester (L-NAME) combination diet for 18 weeks to elicit MetS. The MetS mice were subcutaneously immunized with the ADRQβ-004 vaccine four times to evaluate the therapeutic efficacy in obesity and hypertension and other associated ﻿abnormalities related to MetS by conducting echocardiographic, histological, and biochemical analyses.

**Results:**

The ADRQβ-004 vaccine induced strong antibody production and maintained a high anti-ADR-004 antibody titer in MetS mice. The ADRQβ-004 vaccine improved obesity (*P* < 0.001) and decreased systolic blood pressure (*P* < 0.001). Improvements in dysregulated glucose homeostasis and dyslipidemia resulting from the ADRQβ-004 vaccine were also confirmed. Furthermore, the ADRQβ-004 vaccine attenuated cardiovascular functional (*P* = 0.015) and structural changes (*P* < 0.001), decreased fat accumulation (*P* = 0.012) and inflammation (*P* = 0.050) in the epididymal white adipose tissue, and alleviated hepatic steatosis (*P* = 0.043) involved in MetS. Moreover, the ADRQβ-004 vaccine improved systematic and visceral organs SNS activities in the MetS.

**Conclusion:**

This study demonstrated for the first time that the ADRQβ-004 vaccine targeting α1D-AR improved obesity, hypertension, dyslipidemia, and dysglycemia, and further reduced end-organ damage, which may provide new motivation for MetS research.

**Supplementary Information:**

The online version contains supplementary material available at 10.1007/s10557-022-07418-9.

## Introduction

Metabolic syndrome (MetS) is a common but complex disorder associated with a series of interconnected factors, including abdominal obesity, increased blood pressure, dyslipidemia, and dysglycemia, which increase the risk of cardiovascular disease (CVD) and type 2 diabetes [1]. Numerous studies have evaluated the prevalence of MetS based on different criteria, but all still draw the conclusion that its prevalence is high and rising, and a higher incidence is well recognized as resulting in undesirable outcomes, which also carry a burden of socioeconomic costs as well [2]. Despite therapeutic strategies that involve diet and exercise to promote weight loss and pharmacologic treatment of atherogenic dyslipidemia, hypertension, and hyperglycemia having some efficacy [3], it is not yet known whether MetS can be treated in and of itself [4].

Accumulating evidence from laboratories and human studies indicates that activation of the sympathetic nervous system (SNS) plays an important role in MetS [5]. Hyperinsulinemia, hyperleptinemia, increased circulating adiposity, and obstructive sleep apnea are implicated as involving sympathetic activation, and SNS activation further leads to a series of consequences, including hypertension, insulin resistance (IR), and diastolic dysfunction, which are related to MetS [6]. Focusing therapeutic efforts on sympathetic overactivity, which remains at the core of the pathophysiology of MetS [7], may provide the most overall efficacy. Indeed, the common treatment strategies recommended for patients with MetS, such as diet and exercise to induce weight reduction, are associated with sympathetic inhibition [6]. In addition, pharmacological modulation of the SNS activity is a rational therapeutic strategy for MetS, for example, carvedilol, an α- and β-adrenergic blocker, was associated with an improvement in dyslipidemia and IR [8, 9], but the blood pressure lowering effect of dual α- and β-receptor blockers is less than that of nonselective and β1-selective β-blockers [10]. However, β-blocker drugs, such as metoprolol, atenolol and propranolol, are linked to weight gain and a deterioration of lipid profile and IR, which promote the trend of MetS to developing into type 2 diabetes [11]. Accordingly, targeting α1-adrenergic receptors (α1-ARs) against the SNS seems to be a rational strategy.

α1-ARs, known as G-protein coupled receptors, bind endogenous catecholamine (CA), norepinephrine (NE), and epinephrine, further regulating the SNS; α1-ARs have three subtypes (α1A-AR, α1B-AR, and α1D-AR) and have long been recognized to regulate blood pressure, smooth muscle contraction, and cardiac hypertrophy [12]. α1-adrenergic blockers targeting the SNS show a variety of potential beneficial effects on lipid and glucose metabolism in addition to antihypertension and are widely used in the clinic [13]. Recent research into the molecular processes underlying the metabolic effects suggests that doxazosin, an α1-adrenergic blocker, may promote the production of high-density lipoprotein cholesterol (HDL-C) through gene transcription mechanisms that are independent of its α1-AR antagonist capability [14]. However, α1-adrenergic blockers have some side effects and worsen cerebrovascular events, heart failure, and combined cardiovascular outcomes [15]. On the other hand, stimulation of α1A-AR and α1B-AR mediates several aspects of whole-body and organ-specific metabolism to regulate glucose uptake, gluconeogenesis, glucose breakdown, lipolysis, and fatty acid oxidation for energy production in addition to cardioprotection against ischemia and heart failure, especially α1A-AR [16]. The deletion of functional α1D-AR leads to an antihypertensive effect [17]. Therefore, specific antagonists against the α1D-AR subtype might be more effective therapeutically against MetS by retaining the positive effects of the α1A-AR and α1B-AR subtypes and avoiding negative side effects.

Therapeutic vaccines are in rapid development and are a novel approach for chronic diseases, such as infections, cancer, diabetes, hypertension, and obesity [18], which seem to be a potential and attractive treatment strategy targeting the SNS for MetS. Based on the virus-like particle (VLP), our team invented a hypertension vaccine (ATRQβ-001) [19], a pulmonary hypertension vaccine (ETRQβ-002) [20], a lipid-reduction vaccine (PCSK9Qβ-003) [21], and a specific vaccine targeting α1D-AR (ADRQβ-004) [22], all of which exhibited commonalities such as stable effect, long half-life, and no immune damage to animals. In our earlier study, the ADRQβ-004 vaccine effectively and stably decreased the systolic blood pressure (SBP) of hypertensive animals while not affecting the expression of α1A-AR [22]. This finding suggests the vaccine targeting α1D-AR may exert an antihypertensive effect without affecting the metabolic and cardiac protection of α1A-AR and α1B-AR. Therefore, the ADRQβ-004 vaccine may unlock a new therapeutic strategy for MetS. In this study, MetS mice were elicited by a combination of a high-fat diet (HFD) and N^ω^-nitro-L-arginine methyl ester (L-NAME) as previously described [23] with modification, which recapitulates numerous features of MetS, including obesity, increased blood pressure, dyslipidemia, and dysglycemia. The ADRQβ-004 vaccine was injected into mice to investigate the therapeutic effects by evaluating the above indicators and SNS activity.

## Methods

### Peptide Synthesis and Vaccine Preparation

Peptide synthesis and vaccine preparation were performed as described previously [22]. In brief, the epitope CGITEEAGY (termed ADR-004), which belongs to the second extracellular loop (ECL2) of α1D-AR, was synthesized and validated by GL Ltd. (Shanghai, China) with a purity above 98%. VLP was expressed and purified and then identified using sodium dodecyl sulfate-polyacrylamide gel electrophoresis (SDS-PAGE) and transmission electron microscopy. ADR-004 was covalently conjugated to VLP using a Sulfo-SMCC crosslinker (Thermo Fisher Scientific, Massachusetts, USA) to produce the vaccine called ADRQβ-004. The conjugation rate of the ADRQβ-004 vaccine was analyzed by SDS-PAGE. The concentration of the vaccine was determined using a Bradford protein assay kit (Thermo Fisher Scientific, Massachusetts, USA).

### MetS Experimental Animals

Male C57BL/6N mice aged 6 weeks were purchased from Charles River Laboratories and were randomly divided into three groups: (1) a control group (Con) (n = 12); (2) a HFD + L-NAME-treated group (HFD + L-NAME) (n = 12); and (3) an ADRQβ-004 vaccine group (ADRQβ-004) (n = 12). Two mice were maintained in each cage on a 12-hour light/dark cycle from 6 AM to 6 PM and had unrestricted access to food and water. The control group mice received standard diet and water, while the HFD + L-NAME group mice and the ADRQβ-004 vaccine group mice received HFD (D12492; Research Diet, Inc, New Brunswick, USA (60% of kilocalories from fat (lard))) and L-NAME (1 g/L; TCI, Tokyo, Japan) in drinking water after adjusting the pH to 7.4. HFD food and the L-NAME drinking water were supplied for 18 weeks. Mice in the ADRQβ-004 vaccine group were immunized subcutaneously with the ADRQβ-004 vaccine at weeks 0, 2, 4, and 10 at 200 μg per mouse, while mice in the control and HFD + L-NAME groups were treated with 200 μL of PBS per mouse in the same way. The body weight (BW) of all groups was measured every week. All mice were sacrificed at week 18.

### Tail-Cuff Blood Pressure Recording

Blood pressure (BP) was recorded noninvasively in conscious mice by a computerized tail-cuff system (BP 2010A, Softron, Tokyo, Japan). Animals were placed in an individual dark holder on a temperature-controlled platform (37°C) and recordings were performed under steady-state conditions. Readings were averaged from at least 10 measurements per mouse. The BP measurement was performed by one person who was blinded to the identities of the groups. The BP of all mice was recorded every 2 weeks until the end of the study.

### Enzyme-linked immunosorbent assay (ELISA)

The serum anti-ADR-004 antibody titer was tested by ELISA as previously described [22]. Ninety-six-well plates were treated with 1 μg of peptide each, diluted mice serum was added, and the plates were incubated for 1.5 h. After washing, the plates were incubated with goat anti-mouse IgG peroxidase antibody (1:3000, AntGene, Wuhan, China) for 30 min followed by washing again. The color was assessed using a Bio Teck Epoch microplate reader after adding TMB (Thermo Fisher Scientific, Massachusetts, USA) and terminating with 1 M HCl. The specific peptide antibody titers were detected on days 0, 18, 24, 37, 52, 80, 95, 111, and 126.

### Intraperitoneal Glucose-Tolerance Test (IPGTT) and Insulin-Tolerance Test (ITT)

IPGTTs and ITTs were performed by injection of glucose (2 g/kg BW in saline) and insulin (0.5 IU/kg BW in saline) (Novo Nordisk, Copenhagen, German), respectively, after 6 h of fasting with free access to drinking water. The tail blood glucose level (mmol/L) was measured with a glucometer (Yuwell, Shanghai, China) before (0 min, also the fasting blood glucose) and at 15, 30, 60, 90, and 120 min after glucose and insulin administration. To normalize for differences in basal glucose concentration, these data were displayed as the area under the curve (AUC) values as well. The detection of IPGTT was conducted at weeks 12 and 18, while the ITT was conducted at week 18.

### Biochemical Measurement

The serum for the lipid test and fasting insulin test was obtained after fasting overnight by tail-clipping blood collection at weeks 12 and 18, while other indicators were tested after death. Blood was collected and centrifuged to obtain serum. Serum fasting insulin and lipids, including triglyceride (TG), total cholesterol (TC), low-density lipoprotein cholesterol (LDL-C), and HDL-C, were measured after overnight fasting by using biochemical kits (Quanzhou Ruixin Biological Technology Co., Ltd., Quanzhou, China, and Nanjing Jiancheng Bioengineering Institute, Nanjing, China). Homeostasis model assessment of insulin resistance (HOMA-IR) is an indicator of systemic insulin resistance and is calculated as follows: fasting blood glucose (mmol/L) × fasting insulin (mIU/L)/22.5. Serum nonfasting insulin, leptin, free fatty acid (FFA), N-terminal pro-B type natriuretic peptide (NT-proBNP), CA, and NE were analyzed by ELISA following the manufacturer’s protocol (Quanzhou Ruixin Biological Technology Co., Ltd., Quanzhou, China). TG and TC levels in the liver were determined using commercial kits (Nanjing Jiancheng Bioengineering Institute, Nanjing, China).

### Echocardiography and Doppler Imaging

Transthoracic echocardiography was performed using a VisualSonics Vevo 3100 system equipped with an MX400 transducer (Visual Sonics, Toronto, Canada). Left ventricular ejection fraction (LVEF) and other indices of systolic function were obtained from a short-axis M-mode scan at the midventricular level, as indicated by the presence of papillary muscles, in conscious, gently restrained mice. Apical 4-chamber views were obtained in anesthetized mice for diastolic function measurements using pulsed-wave Doppler imaging at the level of the mitral valve. Anesthesia was induced by 5% isoflurane and confirmed by a lack of response to firm pressure on one of the hind paws. During echocardiogram acquisition, isoflurane was reduced to 1.0–1.5% and adjusted to maintain a heart rate in the range of 500-650 beats per min under body-temperature-controlled conditions. At the end of the procedures, all mice recovered from anesthesia without difficulties. All parameters of left ventricular (LV) structure and function were measured at least three times, and the means are presented.

### Histology and Immunostaining

The heart, liver, epididymal white adipose tissue (eWAT), kidney, and thoracic aorta were collected and divided into two parts. One was flash-frozen in liquid nitrogen and the other was fixed in 4% paraformaldehyde overnight and processed for routine paraffin histology (5-μm sections stained with hematoxylin and eosin, (H&E)). Frozen sections of livers and kidneys were cut into optimal sections for Oil Red O (OR) staining to analyze lipid accumulation. Wheat germ agglutinin (WGA) staining and CD31 staining were used to observe the cross-sectional area of cardiomyocytes and to quantify the capillary density, respectively. F4/80-stained eWAT was analyzed for inflammation by calculating the mean integrated optical density values. Van Gieson staining of the thoracic aorta was analyzed for vascular remodeling. Eight to 10 randomly selected photos from each sample were analyzed by Image-Pro Plus 6.0. The number of animals included in the analysis is labeled on each figure.

### Real-Time Quantitative Polymerase Chain Reaction (qRT-PCR)

Total RNA was extracted from frozen organs using an RNA isolator (Vazyme, Nanjing, China), and then 2 μg of RNA was used to reverse-transcribe into cDNA using the HiScript® III RT SuperMix for qRNA (Vazyme, Nanjing, China). qRT-PCR was performed in duplicate using AceQ® qPCR (Vazyme, Nanjing, China) with specific primers for target sequences. The 2^−ΔΔCt^ relative quantification method, using glyceraldehyde-3-phosphate dehydrogenase (GAPDH) for normalization, was used to estimate the amount of target mRNA in samples, and fold ratios were calculated relative to mRNA expression levels from control samples. The primer sequences used, including forward and reverse sequences for each gene, are shown in the Supplementary Material (Table S2).

### Western Blot

After weighing, the frozen tissues were homogenized by lysis in ice-cold modified RIPA buffer (Beyotime, Shanghai, China) containing protease and phosphatase inhibitors. Then, the homogenates were centrifuged, and the protein concentration of the supernatant was determined using a Bradford protein assay kit (Thermo Fisher Scientific, Massachusetts, USA). Twenty-five micrograms of protein from heart and liver tissue was added to a 15-μL protein loading system, while 50 μg of protein from fat tissue was added to a 20-μL protein loading system. Proteins were separated by 10% PAGE gels (Yamei, Shanghai, China) and transferred to a polyvinylidene fluoride (PVDF) membrane (Millipore, Massachusetts, USA). The blots were probed with the following primary antibodies for a 12-h incubation: anti-tyrosine hydroxylase (TH) monoclonal antibody (1:1000, Santa Cruz Biotechnology, Texas, USA), anti-peroxisome proliferator activated receptor β (PPARβ) (1:1000, Santa Cruz Biotechnology, Texas, USA), and anti-GAPDH monoclonal antibody (1:3000, Proteintech, Wuhan, Hubei). The Western blotting results were quantified using Image Lab software (Bio-Rad Laboratories, CA, USA) and were expressed normalized to the mean of the control group.

### Statistical Analysis

Data are expressed as the mean ± standard deviation (SD). Differences were analyzed by one-way or two-way ANOVA with the Bonferroni-adjusted *t* test for multiple comparisons as appropriate in the experiment when values were normally distributed and the variance was homogeneous, while the Kruskal–Wallis test was used for nonparametric data. For the comparison of two groups, the unpaired 2-tailed Student’s *t* test was used. A value of *P* < 0.05 was considered statistically significant. Statistical analyses were conducted using IBM SPSS Statistics version 25.

## Results

### The ADRQβ-004 Vaccine Improved Obesity and Decreased SBP

**Fig. 1:**
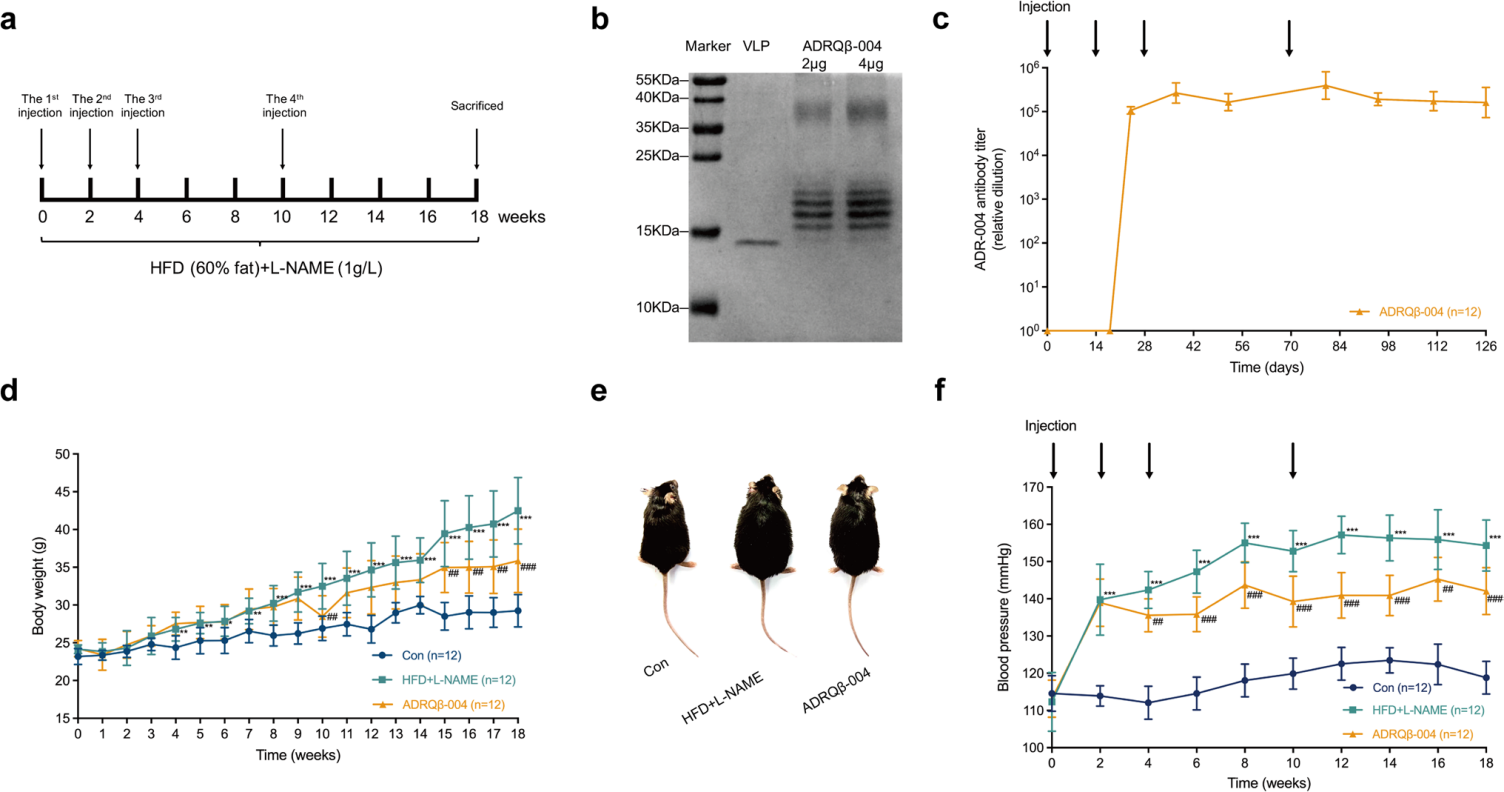
ADRQβ-004 vaccine improved obesity and decreased SBP. **a** Experimental design. C57BL/6N mice were maintained on HFD + L-NAME diet and analyzed at 18 weeks. **b** The sodium dodecyl sulfate-polyacrylamide gel electrophoresis image of the ADRQβ-004 vaccine. **c** ADR-004 specific antibody titers were screened on days 0, 18, 24, 37, 52, 80, 95, 111, and 126. **d** Body weight of mice from different experimental groups during 18 weeks of diet. **e** Representative photographs of body size in different experimental groups. **f** The SBP of each group during the study measured by tail-cuff method. All data are expressed as the mean ± SD. *P < 0.05, **P < 0.01 and ***P < 0.001 vs the control group; ^#^*P* < 0.05, ^##^*P* < 0.01 and ^###^*P* < 0.001 vs the HFD + L-NAME group. ADRQβ-004 indicates the ADRQβ-004 vaccine group; Con, the control group; HFD + L-NAME, the HFD + L-NAME group; HFD, high-fat diet; L-NAME, N^ω^-nitro-L-arginine methyl ester; SBP, systolic blood pressure; VLP, the Qβ virus-like particle

We conducted our experiment on a two-hit murine model fed a HFD and L-NAME combination diet for 18 weeks to explore the effect of the ADRQβ-004 vaccine on MetS (Fig. 1a). First, the ADR-004 epitope was covalently conjugated with VLP (ADRQβ-004 vaccine), and SDS-PAGE showed 1 monomer of VLP coupled to 1 to 5 ADR-004 epitopes (Fig. 1b). The ADRQβ-004 vaccine was subcutaneously injected into mice fed a combination diet. ELISA testing confirmed that the anti-ADR-004 antibody titer rose after the second vaccination (1:80,000 to 1:150,000) and was maintained at a high level thereafter (Fig. 1c). Compared with the control group, the HFD markedly induced an increase in BW after 4 weeks. At individual time points, the BW of the vaccine-treated mice was the same as or lower than that of the HFD + L-NAME group and the difference was not found to be statistically significant between these two groups until week 15. The BW of mice in the ADRQβ-004 vaccine group was significantly different from that of the HFD + L-NAME group mice (ADRQβ-004: 35.9 ± 4.2 g versus HFD + L-NAME: 42.5 ± 4.4 g, *P* < 0.001) at week 18, which developed into obesity (Fig. 1d). The photograph of mice in the different experimental groups showed an obvious discrepancy in body size (Fig. 1e). To evaluate the antihypertensive effect of the ADRQβ-004 vaccine in the two-hit model, BP measurement was conducted every 2 weeks using the tail-cuff method. As a result of L-NAME administration, SBP rose by 33 mmHg on average in the HFD + L-NAME group, decreased in the ADRQβ-004 vaccine group after the second vaccination, and then remained stable; the average decline was 11 mmHg (ADRQβ-004: 140 ± 3 mmHg versus HFD + L-NAME: 151 ± 6 mmHg, *P* < 0.001) (Fig. 1f). Taken together, these data suggested that the ADRQβ-004 vaccine exerts not only a stable antihypertensive role but also a positive impact on obesity.

### The ADRQβ-004 Vaccine Significantly Improved Fasting Blood Glucose and Insulin Resistance and Partly Ameliorated Dyslipidemia

**Fig. 2:**
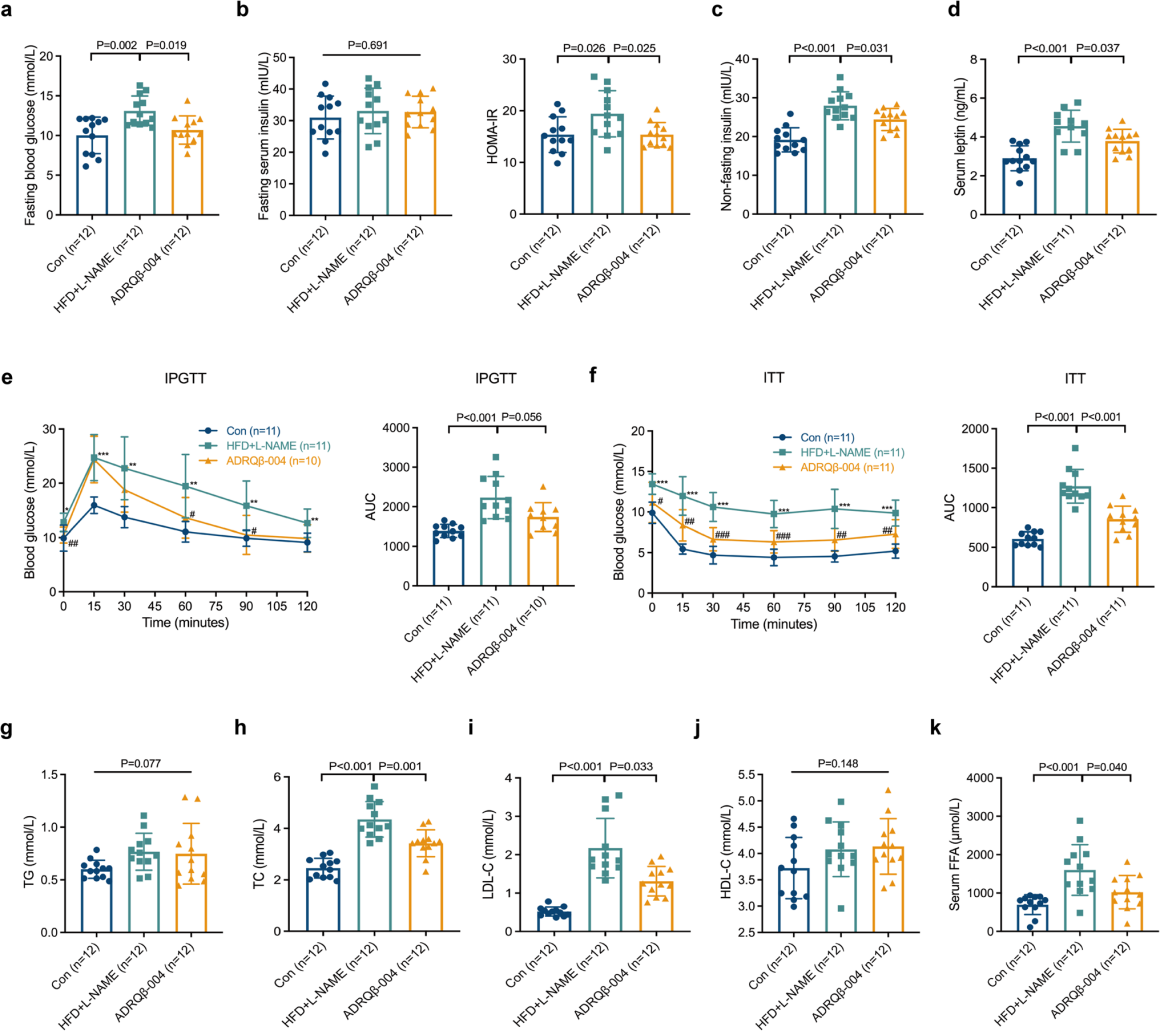
The ADRQβ-004 vaccine significantly improved fasting blood glucose and insulin resistance, and partly ameliorated dyslipidemia. **a**–**c** Fasting blood glucose, fasting serum insulin, HOMA-IR values, and non-fasting serum insulin measured after 18 weeks of diet. **d** The level of serum leptin at the end of the experiment. **e, f** IPGTT and ITT as well as their AUC values of each after 18 weeks of diet. **g**–**j** The serum TG, TC, LDL-C, and HDL-C concentrations after 18 weeks of diet. **k** The level of serum FFA at the end of the experiment. All data are expressed as the mean ± SD. *P < 0.05, **P < 0.01 and ***P < 0.001 vs the control group; ^#^*P* < 0.05, ^##^*P* < 0.01 and ^###^*P* < 0.001 vs the HFD + L-NAME group. ADRQβ-004 indicates the ADRQβ-004 vaccine group; AUC, area under the curve; Con, the control group; FFA, free fatty acid; HDL-C, high-density lipoprotein cholesterol; HFD + L-NAME, the HFD + L-NAME group; HOMA-IR, homeostasis model assessment of insulin resistance; IPGTT, intraperitoneal glucose-tolerance test; ITT, insulin-tolerance test; LDL-C, low-density lipoprotein cholesterol; TC, total cholesterol; TG, triglyceride

We analyzed blood samples at both the 12- and 18-week time points to determine whether the ADRQβ-004 vaccine ameliorates dysregulated glucose homeostasis and dyslipidemia. The HFD + L-NAME group mice developed glycometabolic disorder at week 18, while, the ADRQβ-004 vaccine group mice showed improvement, including a notable decrease in fasting blood glucose (ADRQβ-004: 10.7 ± 1.8 mmol/L versus HFD + L-NAME: 13.1 ± 1.9 mmol/L, *P* = 0.019), nonfasting serum insulin (ADRQβ-004: 24.4 ± 2.8 mIU/L versus HFD + L-NAME: 27.9 ± 3.6 mIU/L, *P* = 0.031), and HOMA-IR level (ADRQβ-004: 15.4 ± 2.3 versus HFD + L-NAME: 19.4 ± 4.5, *P* = 0.025), even though the fasting serum insulin was no different (Fig. 2a-c). The same changes were observed in the serum leptin level at week 18 (ADRQβ-004: 3.79 ± 0.61 ng/mL versus HFD + L-NAME: 4.56 ± 0.82 ng/mL, *P* = 0.037) (Fig. 2d). Subsequently, we performed IPGTTs and ITTs to examine the impact on glucose and insulin tolerance. Compared with the HFD + L-NAME group, mice vaccinated with ADRQβ-004 exhibited a significant decrease in blood glucose values at 60 and 90 min after a glucose challenge, but the AUC values of the IPGTT did not decrease significantly (ADRQβ-004: 1738 ± 364 versus HFD + L-NAME: 2231 ± 534, *P* = 0.056) at week 18 (Fig. 2e), while it was decreased in the 12-week AUC results (ADRQβ-004: 1626 ± 314 versus HFD + L-NAME: 2101 ± 373, *P* = 0.011) (Fig. S2b). In contrast to the HFD + L-NAME group, ADRQβ-004 vaccination also evoked a greater reduction in blood glucose levels at 15, 30, 60, 90, and 120 min following insulin injection, and a reduction in the AUC of ITT accordingly (ADRQβ-004: 854 ± 164 versus HFD + L-NAME: 1271 ± 214, *P* < 0.001) (Fig. 2f).

To investigate the impact of the ADRQβ-004 vaccine on dyslipidemia, we tested serum TG, TC, LDL-C, and HDL-C levels. Compared with the HFD + L-NAME group, a decrease in LDL-C blood level was observed at week 12 (Fig. S2c–e). However, obvious declines in TC (ADRQβ-004: 3.42 ± 0.52 mmol/L versus HFD + L-NAME: 4.35 ± 0.69 mmol/L, *P* = 0.001) and LDL-C (ADRQβ-004: 1.31 ± 0.38 mmol/L versus HFD + L-NAME: 2.17 ± 0.78 mmol/L, *P* = 0.033) were observed in the vaccine group at week 18, and no obvious difference was found in the levels of TG and HDL-C (Fig. 2g–j). Then we measured the alteration of blood FFA level, which paralleled the blood lipid changes (ADRQβ-004: 1022 ± 435 μmol/L versus HFD + L-NAME: 1601 ± 660 μmol/L, *P* = 0.040) (Fig. 2k). Given the dramatic improvement in whole-body glucose and lipid metabolism, we conclude that the ADRQβ-004 vaccine affects dysregulated glucose homeostasis and dyslipidemia.

### The ADRQβ-004 Vaccine Attenuated LV Remodeling and Diastolic Dysfunction

**Fig. 3:**
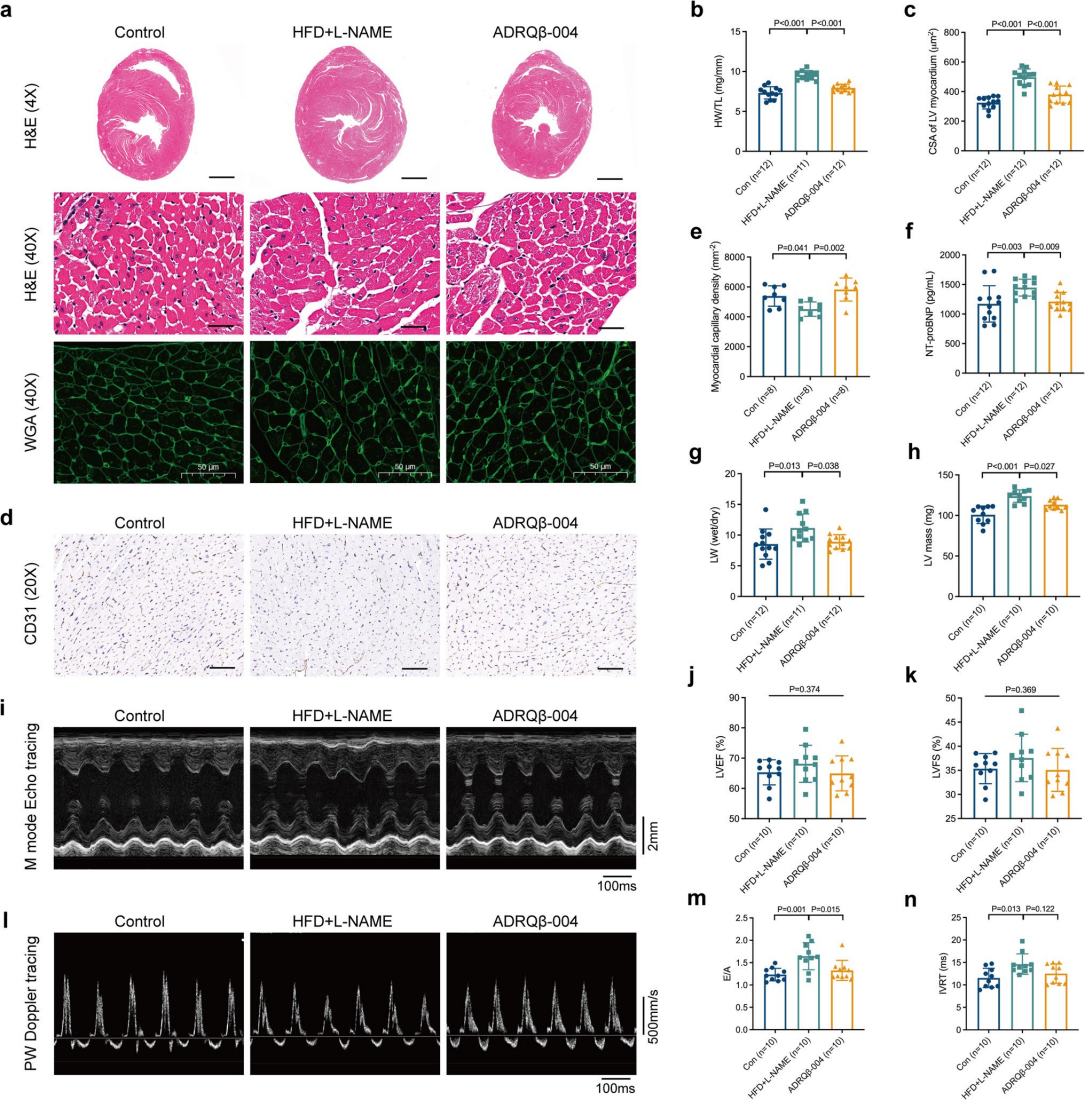
ADRQβ-004 vaccine attenuated LV remodeling and diastolic dysfunction. **a** Representative images of H&E (upper bar = 500 μm, lower bar = 50 μm) and WGA (bar = 50 μm) staining in transversal sections of left ventricle of different experimental groups. **b** Ratio between heart weight and tibia length. **c** Quantification of LV cardiomyocyte CSA. **d** CD31 (bar = 100 μm) staining in transversal sections of LV. **e** Quantification of LV myocardial capillary density. **f** LV mass calculated by echocardiography. **g** Ratio between wet and dry lung weight. **h** The serum level of NT-proBNP. **i** Representative LV M-mode echocardiographic tracings. **j, k** Percentage of LVEF, and LVFS calculated by echocardiography. **l** Representative LV pulsed-wave Doppler tracings. **m, n** Ratio between mitral E wave and A wave, and IVRT calculated by Doppler imaging. All data are expressed as the mean ± SD. ADRQβ-004 indicates the ADRQβ-004 vaccine group; CD31, cluster of differentiation 31; Con, the control group; CSA, cross-sectional area; HFD + L-NAME, the HFD + L-NAME group; H&E, hematoxylin and eosin; IVRT, isovolumic relaxation time; LV, left ventricular; LVEF, left ventricular ejection fraction; LVFS, left ventricular fractional shortening; NT-proBNP, N-terminal pro-B-type natriuretic peptide; WGA, wheat germ agglutinin

MetS increases the risk of CVD; thus, we evaluated cardiac structure and function and the structure of the thoracic aorta. Cardiac LV and cardiomyocyte hypertrophy were observed in mice treated with HFD + L-NAME, while the ADRQβ-004 vaccine effectively attenuated these impacts (ratio between heart weight and tibia length (HW/TL), ADRQβ-004: 7.94 ± 0.47 mg/mm versus HFD + L-NAME: 9.54 ± 0.57 mg/mm, *P* < 0.001; cross-sectional area (CSA) of myocardium, ADRQβ-004: 380 ± 57 μm^2^ versus HFD + L-NAME: 497 ± 55 μm^2^, *P* < 0.001) (Fig. 3a-c). Additionally, myocardial capillary density was lower in the HFD + L-NAME group and higher in the ADRQβ-004-treated group (ADRQβ-004: 5827 ± 768 mm^-2^ versus HFD + L-NAME: 4515 ± 481 mm^-2^, *P* = 0.002) (Fig. 3d, e). The mRNA expression of ANP, BNP, α-MHC, and β-MHC was not significantly different between the HFD + L-NAME group and the ADRQβ-004 vaccine group (Fig. S3a). Animals exposed to both HFD and L-NAME manifested signs of heart failure supported by the NT-proBNP level and lung weight (LW) (wet/dry), indicative of pulmonary congestion, and we observed that these two indicators were modified in the ADRQβ-004 vaccine group (ADRQβ-004: 1211 ± 157 pg/mL versus HFD + L-NAME: 1447 ± 142 pg/mL, *P* = 0.009; ADRQβ-004: 8.9 ± 1.2 versus HFD + L-NAME: 11.2 ± 2.3, *P* = 0.038, respectively) (Fig. 3f, g). Echocardiography showed that the LV mass was also reduced in the vaccine-treated group compared with the HFD + L-NAME group (ADRQβ-004: 113 ± 6 mg versus HFD + L-NAME: 124 ± 8 mg, *P* = 0.027) (Fig. 3h). Short-axis M-mode scans at the midventricular level (Fig. 3i) revealed preservation of LVEF (Fig. 3j) and left ventricular fractional shortening (LVFS) (Fig. 3k) in all groups. The diastolic function index, that is, the ratio between the mitral E wave and A wave (E/A), measured by pulsed-wave Doppler imaging (Fig. 3l), was elevated in the HFD + L-NAME-fed mice and decreased in the ADRQβ-004 vaccine-treated mice (ADRQβ-004: 1.33 ± 0.22 versus HFD + L-NAME: 1.65 ± 0.30, *P* = 0.015) (Fig. 3m), but the results of the isovolumic relaxation time (IVRT) showed no difference between these two groups (ADRQβ-004: 12.5 ± 2.1 ms versus HFD + L-NAME: 14.6 ± 2.3 ms, *P* = 0.122) (Fig. 3n). Other echocardiographic indicators are listed in the Supplementary Material (Table S1). However, vascular structural remodeling (percentage of the medial wall thickness and wall area) induced by the HFD + L-NAME diet was not improved with the addition of ADRQβ-004 (Fig. S4a-e). In summary, the evidence thus far suggests that the ADRQβ-004 vaccine attenuated LV remodeling and diastolic dysfunction.

### The ADRQβ-004 Vaccine Markedly Decreased Fat Mass and Adipocyte Size and Relieved Tissue Inflammation in eWAT

**Fig. 4:**
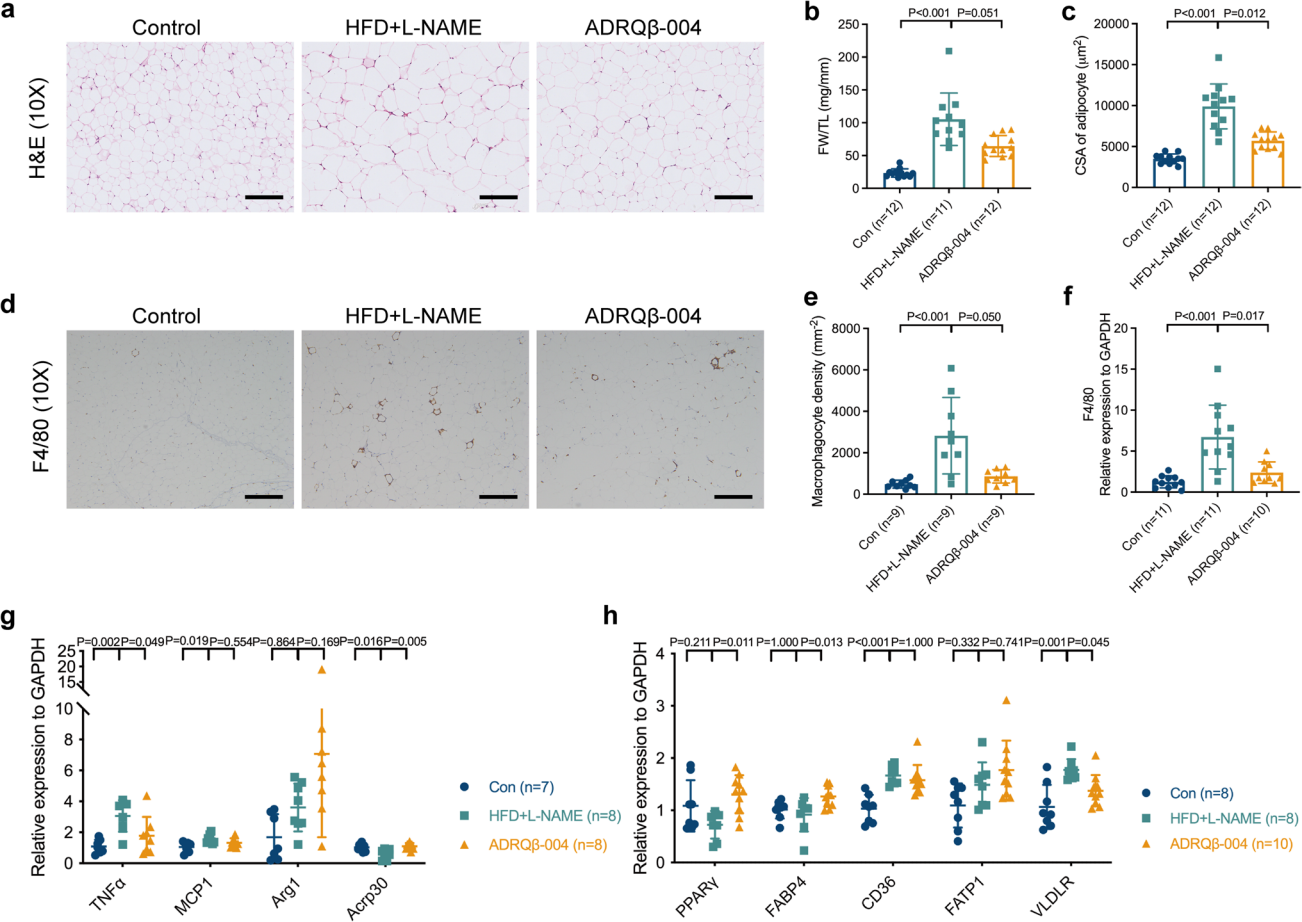
ADRQβ-004 vaccine decreased fat mass and adipocyte size, and relieved tissue inflammation in eWAT. **a** Representative H&E (bar = 200 μm) staining of cross-sections of eWAT isolated from each experimental group. **b** Ratio between eWAT weight and tibia length. **c** Quantification of adipocyte’s CSA. **d** Representative F4/80 (bar = 200 μm) staining of cross-sections of eWAT. **e** Quantification of macrophageocyte density in eWAT. **f** Relative mRNA level of F4/80. **g** Relative mRNA levels of pro- and anti-inflammatory genes. **h** Relative mRNA levels of lipid metabolism. All data are expressed as the mean ± SD. ADRQβ-004 indicates the ADRQβ-004 vaccine group; Arcp30, adiponectin; Arg1, Arginase-1; Con, the control group; CD36, fatty acid translocase; CSA, cross-sectional area; eWAT, epididymal white adipose tissue; FABP4, fatty acid-binding protein 4; FATP1, fatty acid transport protein 1; GAPDH, glyceraldehyde-3-phosphate dehydrogenase; HFD + L-NAME, the HFD + L-NAME group; H&E, hematoxylin and eosin; MCP1, monocyte chemoattractant protein 1; PPARγ, peroxisome proliferator activated receptor γ; TNFα, tumor necrosis factor alpha; VLDLR, very-low-density lipoprotein receptor

We tested the effect of the ADRQβ-004 vaccine on fat accumulation and inflammation. As expected, HFD + L-NAME feeding induced evident adipocyte hypertrophy, in which almost the whole cell was occupied by one large lipid droplet, while the cytoplasm was essentially undetectable (Fig. 4a). The ratio between the eWAT weight and tibia length and the size of the adipocytes of the ADRQβ-004 group mice were lighter and smaller than those of the HFD + L-NAME group mice (ADRQβ-004: 64 ± 16 mg/mm versus HFD + L-NAME: 105 ± 40 mg/mm, *P* = 0.051; ADRQβ-004: 5697 ± 1085 μm^2^ versus HFD + L-NAME: 9899 ± 2748 μm^2^, *P* = 0.012, respectively) (Fig. 4b, c). The chronic inflammatory response of adipose tissue in obesity yielded a potential mechanism underlying systemic IR and type 2 diabetes. We therefore investigated the inflammatory response of eWAT by staining the macrophagocyte activation marker F4/80 (Fig. 4d), and F4/80^+^ cells were immunostained overtly in the HFD + L-NAME group and decreased in the ADRQβ-004 group (ADRQβ-004: 861 ± 320 mm^-2^ versus HFD + L-NAME: 2824 ± 1847 mm^-2^, *P* = 0.050) (Fig. 4e) in parallel with relative mRNA level of F4/80 in eWAT (Fig. 4f). We next tested the expression of another proinflammatory marker genes, namely tumor necrosis factor α (TNFα), which was elevated in the HFD + L-NAME group and decreased in the ADRQβ-004 group (ADRQβ-004: 1.8 ± 1.2 versus HFD + L-NAME: 3.1 ± 1.0, *P* = 0.049), and anti-inflammatory gene expression, including adiponectin (Acrp30), which was decreased in the HFD + L-NAME group and increased in the ADRQβ-004 group (ADRQβ-004: 1.1 ± 0.2 versus HFD + L-NAME: 0.6 ± 0.3, *P* = 0.005) (Fig. 4g). The mRNA expression level of PPARγ, which regulates lipid uptake, storage, insulin-stimulated glucose uptake and inflammation, was reduced in the HFD + L-NAME group and increased in the ADRQβ-004 vaccine group (ADRQβ-004: 1.3 ± 0.4 versus HFD + L-NAME: 0.7 ± 0.3, *P* = 0.011) (Fig. 4h). Elevated very-low-density lipoprotein receptor (VLDLR) expression in adipose tissue and macrophages promotes adipose tissue inflammation and glucose intolerance in obese mice, and we found that the VLDLR mRNA expression level decreased in the ADRQβ-004 vaccine group (ADRQβ-004: 1.4 ± 0.3 versus HFD + L-NAME: 1.8 ± 0.2, *P* = 0.045) (Fig. 4h). Overall, these results reveal that the ADRQβ-004 vaccine is efficient in preventing the increase in fat accumulation and tissue inflammation elicited by HFD + L-NAME feeding.

### The ADRQβ-004 Vaccine Alleviated Hepatic Steatosis Elicited by HFD + L-NAME Feeding

**Fig. 5:**
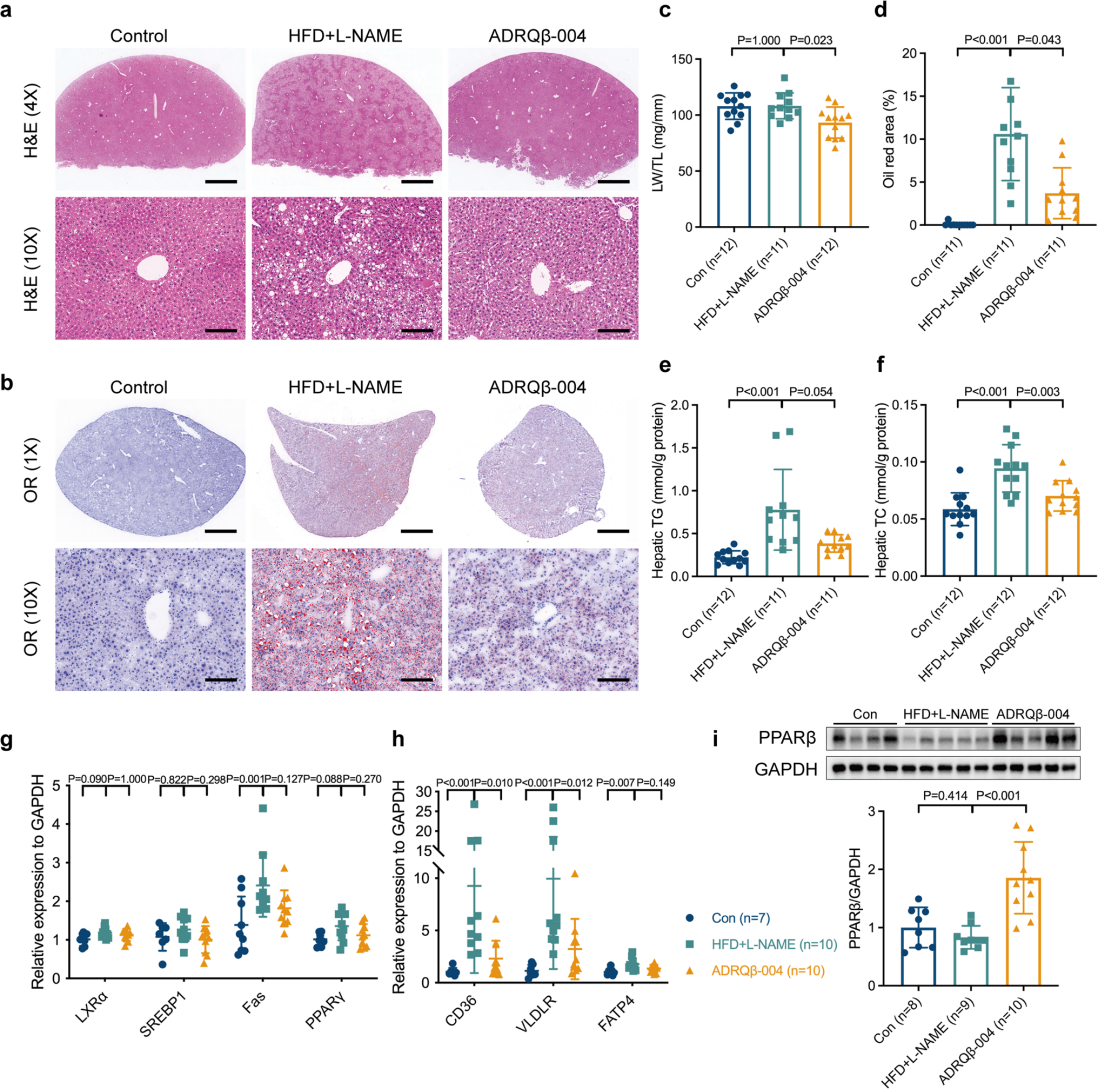
ADRQβ-004 vaccine alleviated hepatic steatosis elicited by HFD + L-NAME-feeding. **a, b** Representative images of H&E (upper bar = 500 μm, lower bar = 20 μm) staining and Oil Red O (upper bar = 500 μm, lower bar = 20 μm) staining of liver section from each group. **c** Ratio between liver weight and tibia length. **d** Percentage of OR area. **e, f** The hepatic TG and TC contents. **g, h** Gene expressions of lipogenic transcription factors, lipid uptake, and transport, respectively. **i** The protein expression level of PPARβ in the liver. All data are expressed as the mean ± SD. ADRQβ-004 indicates the ADRQβ-004 vaccine group; CD36, fatty acid translocase; Con, the control group; Fas, fatty acid synthase; FATP4, fatty acid transport protein 4; GAPDH, glyceraldehyde-3-phosphate dehydrogenase; H&E, hematoxylin and eosin; HFD + L-NAME, the HFD + L-NAME group; LXRα, liver X receptor α; OR, Oil Red O; PPAR, peroxisome proliferator activated receptor; TC, total cholesterol; TG, triglyceride; SREBP1, sterol regulatory element-binding protein; VLDLR, very-low-density lipoprotein receptor

Regarding the liver as a major organ where lipid uptake and utilization occur, H&E and OR staining of liver sections were performed, in which we found that the HFD + L-NAME group exhibited a substantial amount of lipid deposition, which was markedly alleviated in the ADRQβ-004 group (Fig. 5a, b). The ratio between the liver weight and tibia length (LW/TL) showed a difference between the HFD + L-NAME group and the ADRQβ-004 group (ADRQβ-004: 93 ± 14 mg/mm versus HFD + L-NAME: 108 ± 12 mg/mm, *P* = 0.023) (Fig. 5c). The quantification of OR staining showed that the ADRQβ-004 vaccine markedly decreased the formation of fatty liver (ADRQβ-004: 3.7 ± 3.0% versus HFD + L-NAME: 10.6 ± 5.4%, *P* = 0.043) (Fig. 5d). Biochemical analysis of liver TG and TC contents showed results that were consistent with the morphological evaluation (ADRQβ-004: 0.39 ± 0.10 mmol/g protein versus HFD + L-NAME: 0.78 ± 0.47 mmol/g protein, *P* = 0.054; ADRQβ-004: 0.07 ± 0.01 mmol/g protein versus HFD + L-NAME: 0.09 ± 0.02 mmol/g protein, *P* = 0.003, respectively) (Fig. 5e, f). Gene expression levels of the lipogenic transcription factors liver X receptor α (LXRα) and sterol regulatory element-binding protein 1 (SREBP1), as well as their downstream targets fatty acid synthase (Fas) and PPARγ were measured. Uniquely, Fas significantly increased in the HFD + L-NAME group but was not decreased in the ADRQβ-004 group (ADRQβ-004: 1.8 ± 0.5 versus HFD + L-NAME: 2.4 ± 0.8, *P* = 0.127) (Fig. 5g). Higher mRNA expression levels related to lipid uptake and transport, such as CD36 (ADRQβ-004: 2.3 ± 1.7 versus HFD + L-NAME: 9.3 ± 8.3, *P* = 0.010) and VLDLR (ADRQβ-004: 3.2 ± 2.9 versus HFD + L-NAME: 9.9 ± 8.6, *P* = 0.012), but not fatty acid transport protein 4 (FATP4) (ADRQβ-004: 1.4 ± 0.4 versus HFD + L-NAME: 1.8 ± 0.6, *P* = 0.149), were significantly decreased by ADRQβ-004 administration (Fig. 5h). The protein expression of PPARβ, which is implicated in fatty acid metabolism and plays a key role between α1-AR and multiorgan energetic reprogramming function [24, 25], was increased in the ADRQβ-004 vaccine group compared with the HFD + L-NAME group and the control group (ADRQβ-004: 1.9 ± 0.6 versus HFD + L-NAME: 0.8 ± 0.2, *P* < 0.001). Thus, the ADRQβ-004 vaccine alleviated hepatic steatosis.

### The ADRQβ-004 Vaccine Improved Systematic Sympathetic Activity and that of Organs

**Fig. 6:**
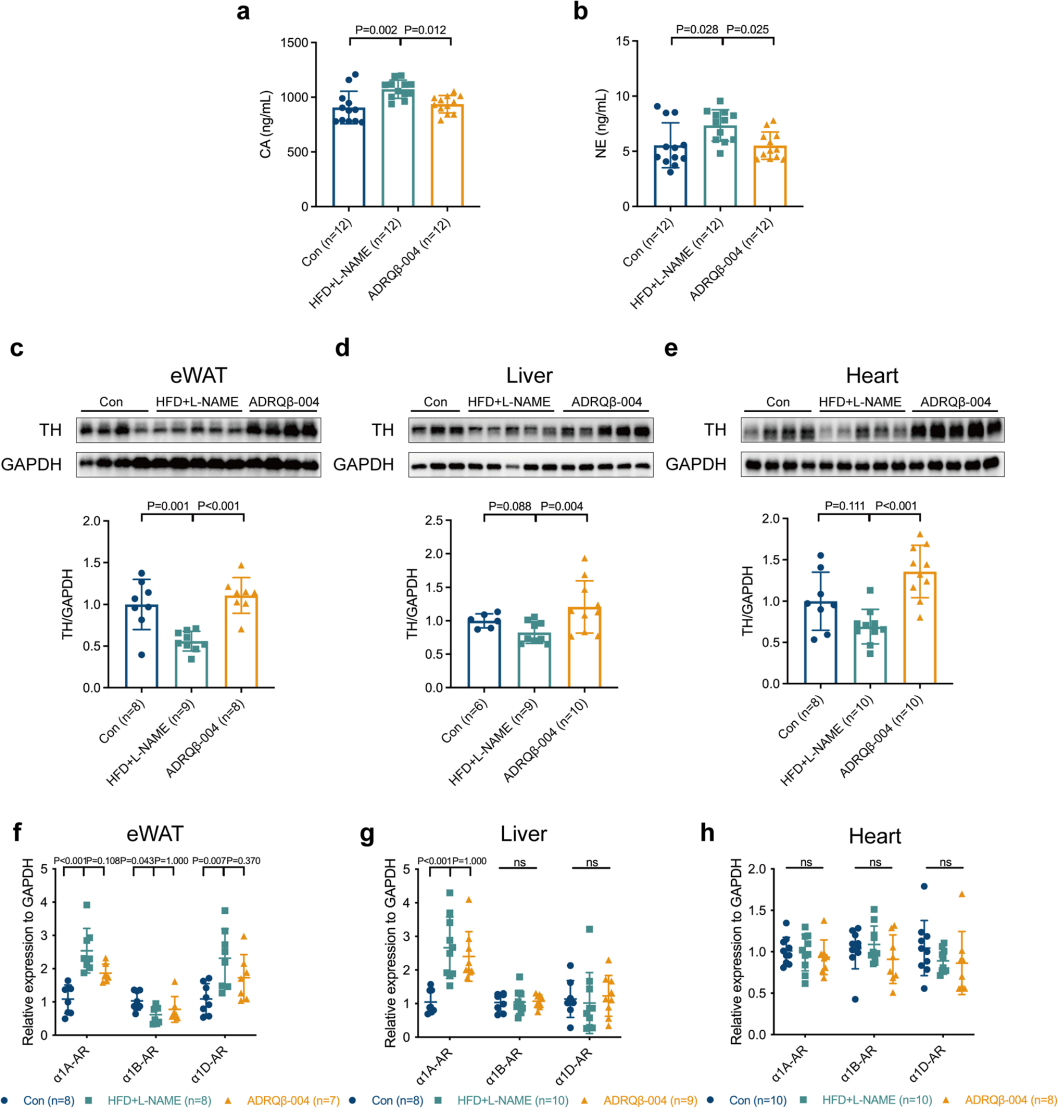
ADRQβ-004 vaccine regulated systematic sympathetic activity and that of end-organs. **a, b** The blood CA and NE concentrations. **c–e** The protein expression level of TH in eWAT, liver, and heart respectively. **f–h** mRNA levels of α1-AR subtypes in eWAT, liver, and heart. Data are expressed as mean ± SD. ADRQβ-004 indicates the ADRQβ-004 vaccine group; α1-AR, α1-adrenergic receptor; CA, catecholamine; Con, the control group; eWAT, epididymal white adipose tissue; GAPDH, glyceraldehyde-3-phosphate dehydrogenase; HFD + L-NAME, the HFD + L-NAME group; NE, norepinephrine; TH, tyrosine hydroxylase

To understand how the ADRQβ-004 vaccine affected MetS, we assessed blood CA and NE concentrations and found that the HFD + L-NAME group had higher CA and NE levels, while the ADRQβ-004 group had lower levels (ADRQβ-004: 936 ± 80 ng/mL versus HFD + L-NAME: 1073 ± 83 ng/mL, *P* = 0.012; ADRQβ-004: 5.52 ±1.24 ng/mL versus HFD + L-NAME: 7.35 ± 1.43 ng/mL, *P* = 0.025, respectively) (Fig. 6a, b). The protein expression of TH in the visceral organs, including eWAT (ADRQβ-004: 1.1 ± 0.2 versus HFD + L-NAME: 0.6 ± 0.1, *P* < 0.001) (Fig. 6c), liver (ADRQβ-004: 1.2 ± 0.4 versus HFD + L-NAME: 0.8 ± 0.2, *P* = 0.004) (Fig. 6d), and heart (ADRQβ-004: 1.3 ± 0.3 versus HFD + L-NAME: 0.7 ± 0.2, *P* < 0.001) (Fig. 6e), was decreased in the HFD + L-NAME group but increased in the ADRQβ-004 vaccine group. We examined the expression of receptors at the mRNA level to determine the changes in α1-AR subtypes in MetS. In eWAT, α1A-AR and α1D-AR were both found to increase in the HFD + L-NAME group but there were no differences between the ADRQβ-004 vaccine group and the HFD + L-NAME group (ADRQβ-004: 1.9 ± 0.3 versus HFD + L-NAME: 2.5 ± 0.7, *P* = 0.108; ADRQβ-004: 1.7 ± 0.7 versus HFD + L-NAME: 2.3 ± 0.9, *P* = 0.370, respectively) (Fig. 6f). However, analysis of the gene expression of α1-AR subtypes in the liver and heart showed that the mRNA levels were not obviously different between the HFD + L-NAME group and the ADRQβ-004 vaccine group (Fig. 6g, h). In conclusion, the ADRQβ-004 vaccine modulated the systematic and visceral organs SNS activities in the MetS.

## Discussion

MetS, which has a complex relationship with the SNS and is linked to a variety of interrelated variables, is a prevalent but complicated condition with few effective treatment options. This study shows for the first time that immunotherapy against SNS targeting α1D-AR successfully improved obesity, hypertension, dyslipidemia, and dysglycemia and further reduced end-organ damage.

Using a HFD + L-NAME paradigm for 18 weeks, we produced MetS in this study with obvious obesity and hypertension, both of which were mitigated by ADRQβ-004 vaccination. The ADRQβ-004 vaccine attenuated the structural and functional abnormalities of the heart, which can be explained by the decline in BP [22]. We also found that fat accumulation and inflammation related to IR and type 2 diabetes [26] were improved in the vaccine-treated group, which might be associated with the decrease in blood leptin level. Blood leptin level is inversely correlated with obesity and body fat level [27], and exhibits a “leptin resistance” phenomenon [28], which is thought to play a key role in the relationship between obesity and MetS [29]. The liver of mice on the combination diet developed steatosis, and hepatic steatosis was improved in the ADRQβ-004 vaccine group, which might be related to lower blood level of FFA, which serves as a fuel for the gluconeogenesis and lipogenesis in the liver [30]. Furthermore, the blood indicators reflecting glucose and lipid metabolism were dysregulated in the HFD + L-NAME group and were alleviated by ADRQβ-004 vaccination except for blood TG and HDL-C. However, elevated TG and reduced HDL-C are part of the criteria for the clinical diagnosis of MetS [1], so this became a limitation of this study in terms of fully recapitulating MetS. This might be related to the progress of the HFD model in eliciting dysregulated glucose and lipid metabolism. HFD-fed mice are continually exposed to a positive feedback loop with an increasing level of pathology starting with visceral obesity at first, then aberrant metabolic function and IR, and thereafter increasing TG and decreasing HDL-C levels [31]. Thus, an 18-week HFD may not be long enough to develop blood TG and HDL-C changes. A 16-month HFD (D12492, Research Diet Inc.) experiment showed that there was no difference between the normal and obese mice groups in blood TG level [32]. Hence, lengthening the HFD time and modifying the HFD type may be priorities to improve our study. In addition, our observation of blood insulin concentrations in the fasting and nonfasting states showed different results. The measurement of fasting and nonfasting circulating glucose and insulin is the primary screen to evaluate glucose homeostasis. A 5- to 6-hour fast is often used in mice to determine whether fasting glucose levels are normal. Since they tend to nibble throughout the day, mice are rarely in a “true” fasted state [33]. Therefore, measuring glucose and insulin concentrations in the nonfasting state may be informative as well. Although we did not see a significant difference in fasting blood insulin concentration among these three groups, the results of HOMA-IR, which is regarded as an index of insulin resistance, did show a significant difference between the ADRQβ-004 vaccine group and the HFD + L-NAME group (*P* = 0.025). We assumed that the increase in nonfasting blood insulin levels in the HFD + L-NAME group was secondary to insulin resistance as a consequence of feeding-induced increased insulin secretory demand [34], while in the ADRQβ-004 vaccine group, improved insulin resistance showed a low level of blood insulin in the nonfasting state.

What warrants attention is the impact of the ADRQβ-004 vaccine on systematic sympathetic activity and the activity of organs. Regarding the sympathetic nervous system’s role in obesity, there have been two main conflicting theories. The initial theory, put forth by Landsberg [35], claims that obesity results in IR and hyperinsulinemia, which in turn activates the SNS. The other, proposed by Bray [36], claims that the SNS is underactive in the majority of obese states, which lowers thermogenesis and predisposes individuals to obesity. In this study, we found that the HFD + L-NAME group had higher blood CA and norepinephrine levels, both of which were reduced by ADRQβ-004 vaccination. However, TH, serving as the rate-limiting enzyme of CA biosynthesis [37], was lower in the HFD + L-NAME group and higher in the vaccine-treated group by western blot analysis of eWAT. This result was in line with Friedman and colleagues’ study [38], which found that 4-month HFD-fed mice showed a significant decrease in sympathetic innervation in adipose depots and became increasingly leptin-insensitive. The higher TH expression in eWAT might mean more sympathetic innervation or more stimulation of sympathetic inputs to induce a local lipolytic response, depletion of white adipose mass, and leptin resistance [39]. In the liver and heart, TH protein expression tended to decline in the HFD + L-NAME group and rose obviously in the ADRQβ-004 vaccine group. In general, the systematic SNS activity and the local tissue SNS activities should remain synchronized, i.e., they activate or inactivate simultaneously, but sometimes they are completely separated. In line with several physiological and pathological conditions, the regional heterogeneity of sympathetic nervous activation can be seen in obese individuals, where stimulation of the sympathetic outflow to one organ may be accompanied by normal or diminished sympathetic tone in other organs [40]. The cardiac sympathetic outflow can be unchanged or activated with overfeeding in experimental animals, in contrast with the cardiac sympathetic inhibition seen in human obesity [41]. The difference in SNS activities might be related to blunted local sympathetic responses [42] or to depletion of local CA [43] due to prolonged SNS activation, which contribute to deficient thermogenesis, positive energy balance, and increased weight gain.

The CGITEEAGY peptide that we screened, also known as ADR-004 peptide, specifically belongs to ECL2 of human α1D-AR, which was determined using the Protein BLAST tool on the NCBI website. ECL2 of α1-AR can also alter the subtype selectivity of ligands acting as a selectivity filter [44]. The autoantibodies display various functions for the same receptor, including activation, inhibition, and antigen-antibody binding without signal transduction activity [45]. As a ligand of α1-AR, NE is released in the synaptic cleft by exocytosis of vesicles in the sympathetic nerve terminals. It can either act on postsynaptic receptors on the target cell or on presynaptic receptors to modulate nerve activity [12] by negative feedback control [46]. Although α1D-AR is a postsynaptic receptor, autoantibodies against α1D-AR specifically target the ECL2, exerting an inhibitory effect, and changes in the local NE concentration might result in negative feedback by presynaptic receptors to decrease TH enzyme activity. In addition, the altered responsiveness of SNS could be caused by downregulated or upregulated expression of α1-AR subtypes [42, 47]. Our results showed changes in α1-AR subtypes at the mRNA level, especially in eWAT, which plays a key role in obesity [48, 49]. Even though ADRQβ-004 treatment decreased the mRNA abundance for the α1-AR subtypes compared with the HFD + L-NAME group, we cannot rule out the existence of discrepancies in mRNA and protein levels [42]. Furthermore, since α1-AR plays an important role in mediating the SNS peripheral response, we cannot solely attribute the impaired response to changes in receptor expression. Rather, it is likely that MetS-induced signaling pathways have been altered over time, leading to the tissue heterogeneity seen in adrenoceptor expression, which has been found in diabetes [50] and needs to be explored in further studies about MetS.

While the findings of our trial are encouraging, it remains unclear how the ADRQβ-004 vaccine against SNS targeting α1D-AR can help to improve MetS and attenuate its adverse outcomes. However, the decline in systematic sympathetic activity and the increase in that in organs raised some possibilities that the metabolic benefits of selectively inhibiting α1D-AR might be due to modulating blunted sympathetic responses, which needs further assessments to confirm; or that it might be due to the modulation of α1-AR subtypes in end-organs but does not affect the function of α1A-AR, which has metabolic and cardiac-protective benefits verified in several transgenic and knockout animal models; or that it might be due to the potential effects of α1D-AR in metabolism that are still undiscovered and require more attention and research. This exploration may also provide novel motivation for α1-AR research.

In summary, our investigation revealed that the ADRQβ-004 vaccine markedly improved obesity, hypertension, dyslipidemia, and dysglycemia and further reduced end-organ damage. Additionally, the ADRQβ-004 antibody had a substantially longer half-life than oral medications, which may offer superior benefits for patients compliance. The ADRQβ-004 vaccine targeting the SNS appears to be a sensible and appealing method for MetS treatment.

### Supplementary Information

ESM 1 (DOCX 9411 kb)

## Data Availability

Most of the data described in the article are contained within the article or in the supporting information. Further information and requests for resources and reagents should be directed to and will be fulfilled by Zhihua Qiu (qiu_zhihua512@163.com) and Zihua Zhou (zzhua2001@163.com).

## Abbreviations

MetS: metabolic syndrome
CVD: cardiovascular disease
SNS: sympathetic nervous system
IR: insulin resistance
α1-ARs: α1-adrenergic receptors
CA: catecholamine
NE: norepinephrine
HDL-C: high-density lipoprotein cholesterol
VLP: virus-like particles
HFD: high-fat diet
L-NAME: N^ω^-nitro- L -arginine methyl ester
SDS-PAGE: sodium dodecyl sulfate polyacrylamide gel electrophoresis
BW: body weight
BP: blood pressure
ELISA: enzyme-linked immunosorbent assay
IPGTT: intraperitoneal glucose-tolerance test
ITT: insulin-tolerance test
AUC: area under the curve
TG: triglyceride
TC: total cholesterol
LDL-C: low-density lipoprotein cholesterol
HOMA-IR: homestasis model assessment of insulin resistance
FFA: free fatty acid
NT-proBNP: N-terminal pro-B type natriuretic peptide
LVEF: left ventricular ejection fraction
LV: left ventricular
eWAT: epididymal white adipose tissue
H&E: hematoxylin and eosin
OR: oil red O
WGA: wheat germ agglutinin
qRT-PCR: real-time quantitative polymerase chain reaction
GAPDH: glyceraldehyde-3-phosphate dehydrogenase
PVDF: polyvinylidene fluoride
TH: tyrosine hydroxylase
SD: standard deviation
HW/TL: ratio between heart weight and tibia length
CSA: cross-sectional area
LW: lung weight
LVFS: left ventricular fractional shortening
E/A: ratio between mitral E wave and A wave
IVRT: isovolumic relaxation time
TNFα: tumor necrosis factor α
Acrp30: adiponectin
PPARγ: peroxisome proliferator activated receptor γ
VLDLR: very-low-density lipoprotein receptor
LW/TL: ratio between liver weight and tibia length
LXRα: liver X receptor α
SREBP1: sterol regulatory element-binding protein 1
Fas: fatty acid synthase
FATP4: fatty acid transport protein 4
ECL2: second extracellular loop
