# Supplementary material for: Vaccine Targeting Alpha 1D-Adrenergic Receptor Improved Metabolic Syndrome in Mice
Source: Cardiovasc Drugs Ther. 2023 Jan 19;38(3):539–54. doi: 10.1007/s10557-022-07418-9 (PMC11101575; doi:10.1007/s10557-022-07418-9)
Supplement: Supplementary file 1 — (DOCX 9411 kb) [file 10557_2022_7418_MOESM1_ESM.docx]

**Article Title**: Vaccine Targeting Alpha 1D-Adrenergic Receptor Improved Metabolic Syndrome in Mice

**Journal Name**: Cardiovascular Drugs and Therapy

**Authors**: Xin Li, MSc;^1,2,3+^ Wenrui Ma, PhD;^1,2,3+^ Chang Li, PhD;^1,2,3^ Dingyang Shi, PhD;^1,2,3^ Wenlong Kuang, MSc; ^1,2,3^ Jiacheng Wu, MSc;^1,2,3^ Yuhua Liao, MD^1,2,3^, Zhihua Qiu, PhD;^1,2,3^* Zihua Zhou, PhD;^1,2,3^*

^1^Department of Cardiology, Union Hospital, Tongji Medical College, Huazhong University of Science and Technology, Wuhan 430022, China;

^2^Hubei Key Laboratory of Biological Targeted Therapy, Union Hospital, Tongji Medical College, Huazhong University of Science and Technology, Wuhan, 430022, China;

^3^Hubei Provincial Engineering Research Center of Immunological Diagnosis and Therapy for Cardiovascular Diseases, Union Hospital, Tongji Medical College, Huazhong University of Science and Technology, Wuhan, 430022, China

**Addresses for Correspondence:**

Zhihua Qiu, PhD, and Zihua Zhou, PhD

Department of Cardiology, Institute of Cardiology, Hubei Key Laboratory of Biological Targeted Therapy, Union Hospital, Tongji Medical College, Huazhong University of Science and Technology

No. 1277 Jiefang Avenue, Wuhan 430022, China

Email: qiu_zhihua512@163.com or zzhua2001@163.com


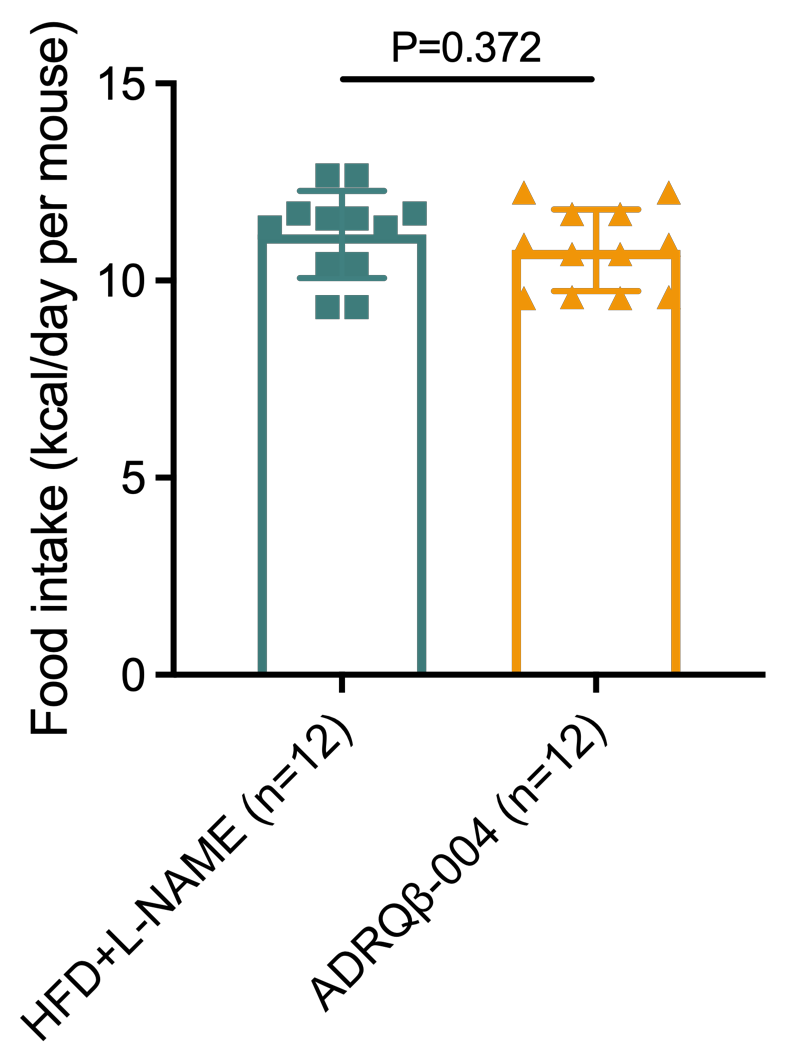


**Supplementary Fig.1 The food intake of the HFD + L-NAME group and the ADRQβ-004 vaccine group.** Data are expressed as the mean ± SD. ADRQβ-004 indicates the ADRQβ-004 vaccine group; HFD + L-NAME, the HFD + L-NAME group.


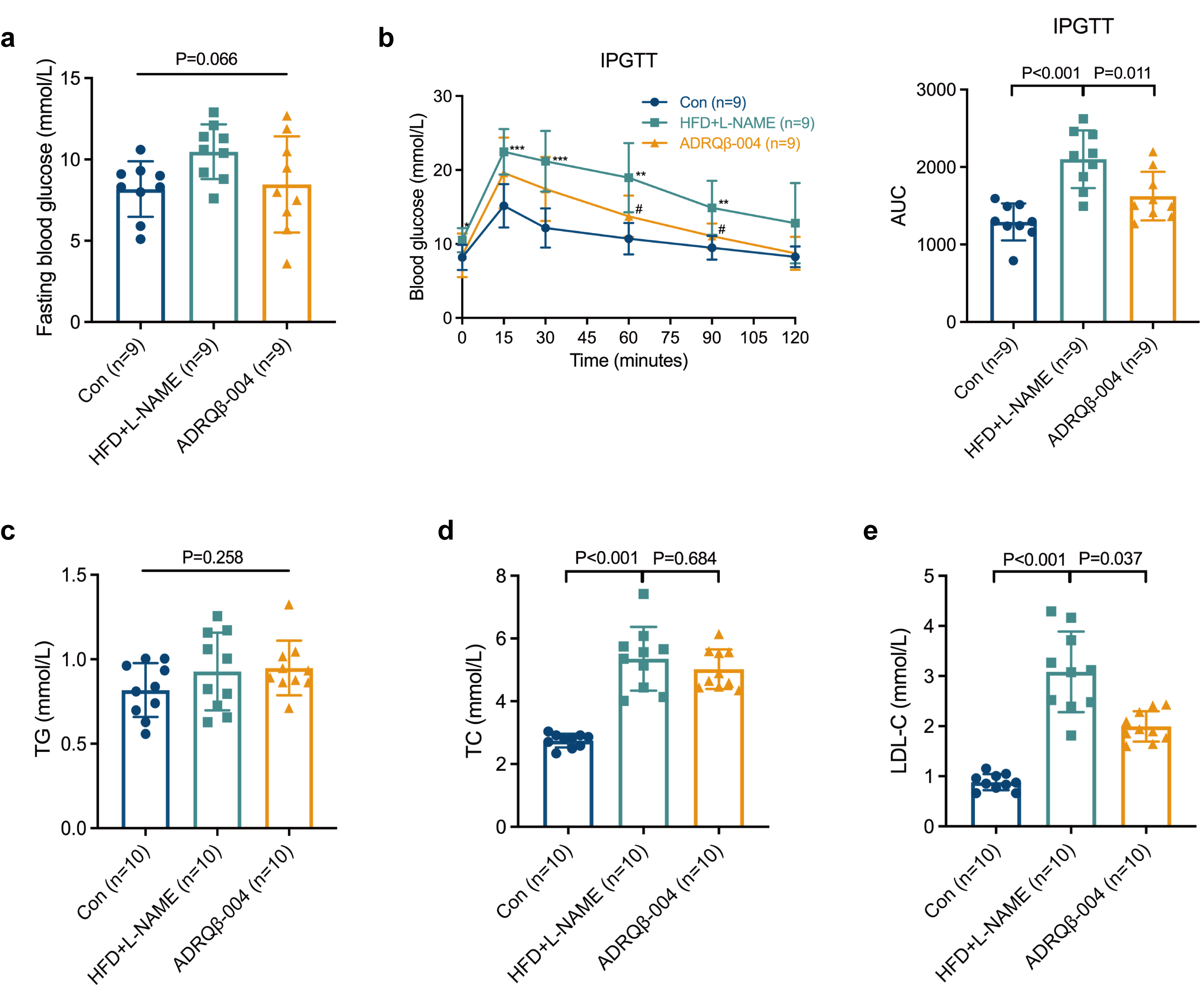


**Supplementary Fig. 2** **ADRQβ-004 vaccine ameliorated dysregulated glucose homeostasis and dyslipidemia at week 12.** **a** and **b**, Fasting blood glucose and IPGTT as well as AUC values after 12 weeks of diet. **c** - **e,** The serum TG, TC, and LDL-C concentrations after 18 weeks of diet. Data are expressed as mean ± SD. *P<0.05, **P<0.01 and ***P<0.001 vs the control group; ^#^P<0.05, ^##^P<0,01 and ^###^P<0.001 vs the HFD + L-NAME group. ADRQβ-004 indicates the ADRQβ-004 vaccine group; AUC, area under the curve; Con, the control group; HFD + L-NAME, the HFD + L-NAME group; LDL-C, low-density lipoprotein cholesterol; TC, total cholesterol; TG, triglyceride.


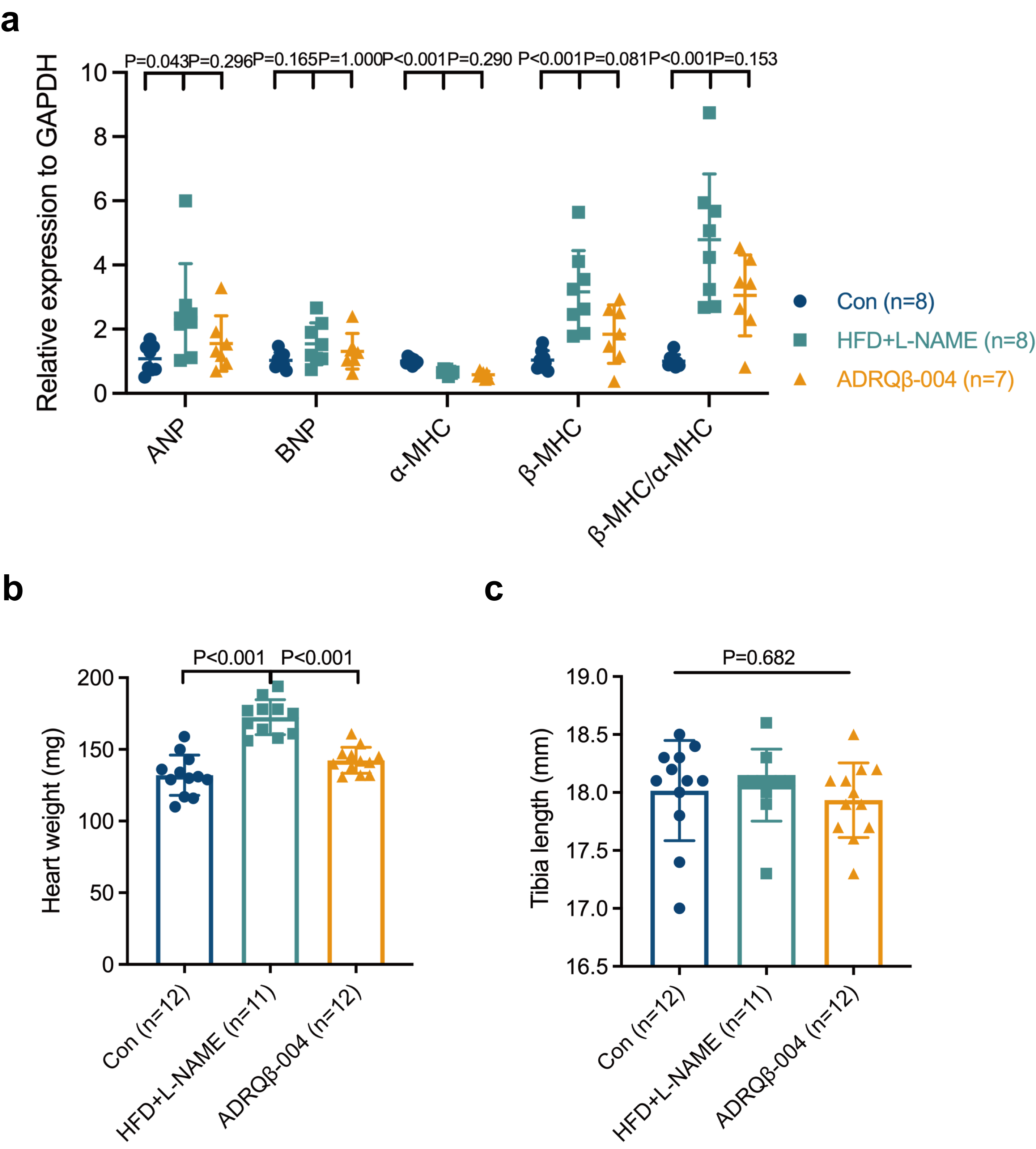


**Supplementary Fig. 3** **mRNA expression level of cardiac hypertrophy, and heart weight and tibia length of all mice groups.** **a**, mRNA expression level of cardiac hypertrophy. **b**, Heart weight of all mice groups. **c**, Tibia length of all mice groups. Data are expressed as mean ± SD. ADRQβ-004 indicates the ADRQβ-004 vaccine group; α-MHC, alpha-myosin heavy chain; ANP, atrial natriuretic peptide; BNP, brain natriuretic peptide; β-MHC, beta-myosin heavy chain; Con, the control group; HFD + L-NAME, the HFD + L-NAME group.


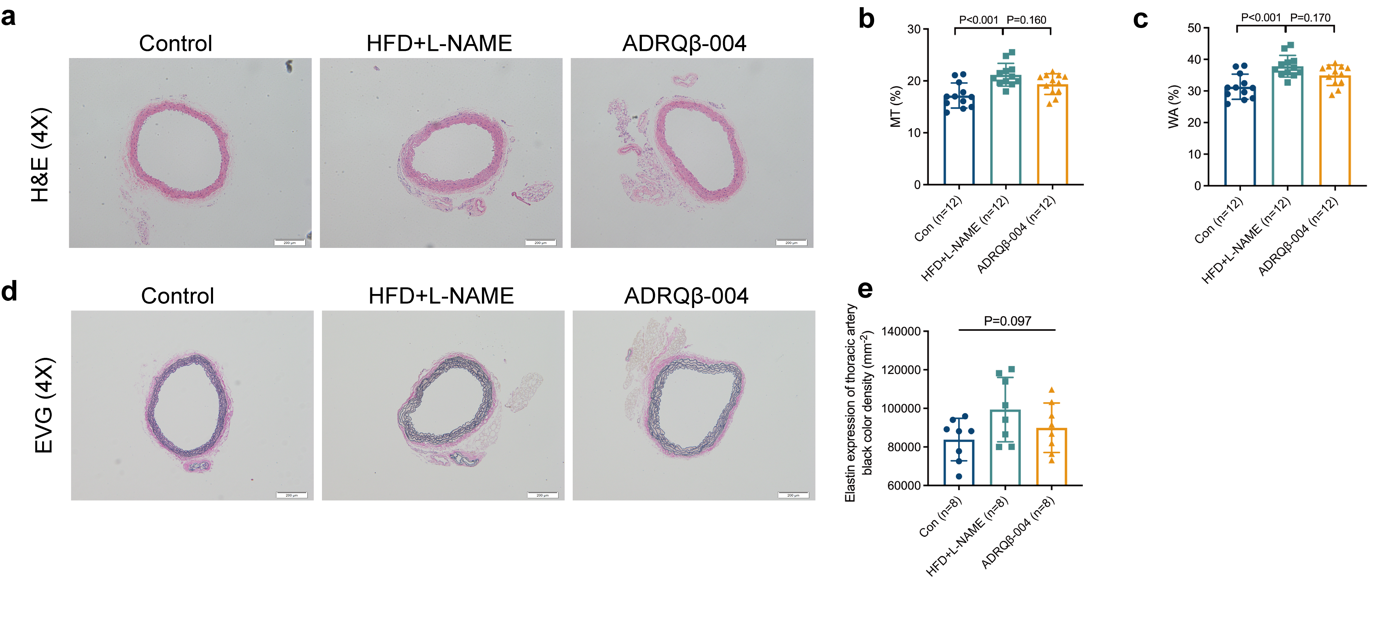


**Supplementary Fig. 4** **Structural remodeling of thoracic aorta.** **a,** Representative photograph of H&E (bar = 200 μm) staining of thoracic aorta. **b** and **c,** The MT% and WA% of vessels. **d,** Representative photographs of EVG (bar = 200 μm) staining. **e,** Quantification of elastin expression. All data are expressed as mean ± SD. ADRQβ-004 indicates the ADRQβ-004 vaccine group; Con, the control group; EVG, Verhoeff’s Van Gieson; H&E, hematoxylin and eosin.; HFD + L-NAME, the HFD + L-NAME group; MT, medial wall thickness; WA, wall area.

| **Supplementary Table 1. Echocardiographic parameters in the different experimental groups of mice.** | | | |
| --- | --- | --- | --- |
| **Parameters** | **Con**  **(n=10)** | **HFD + L-NAME (n=10)** | **ADRQβ-004**  **(n=10)** |
| HR, bpm | 601.74 ± 37.46 | 560.55 ± 63.39 | 590.52 ± 53.38 |
| LV mass, mg | 100.88 ± 10.79# | 123.94 ± 7.63 | 113.39 ± 6.09***** |
| LVPWs, mm | 1.24 ± 0.13***** | 1.44 ± 0.15 | 1.36 ± 0.18 |
| LVPWd, mm | 0.81 ± 0.15***** | 0.99 ± 0.08 | 0.97 ± 0.16 |
| LVAWs, mm | 1.51 ± 0.16# | 1.71 ± 0.13 | 1.64 ± 0.18 |
| LVAWd, mm | 0.96 ± 0.10# | 1.13 ± 0.09 | 1.06 ± 0.09 |
| LVIDs, mm | 2.48 ± 0.15 | 2.32 ± 0.24 | 2.37 ± 0.21 |
| LVIDd, mm | 3.83 ± 0.24 | 3.71 ± 0.18 | 3.65 ± 0.25 |
| LVESV, μL | 21.94 ± 3.09 | 18.82 ± 4.86 | 19.74 ± 4.24 |
| LVEDV, μL | 63.63 ± 9.18 | 58.69 ± 6.59 | 56.57 ± 8.46 |
| SV, μL | 41.69 ± 7.46 | 39.87 ± 4.59 | 36.83 ± 6.88 |
| CO, mL/min | 25.06 ± 4.62 | 22.37 ± 3.83 | 21.90 ± 5.38 |
| LVEF, % | 65.31 ± 4.16 | 68.13 ± 6.12 | 64.97 ± 5.79 |
| LVFS, % | 35.35 ± 3.12 | 37.57 ± 4.93 | 35.09 ± 4.45 |
| IVRT, ms | 11.56 ± 2.14***** | 14.62 ± 2.28 | 12.51 ± 2.15 |
| E/A | 1.23 ± 0.14# | 1.65 ± 0.30 | 1.33 ± 0.22***** |

Data are expressed as mean ± SD. ADRQβ-004 indicates the ADRQβ-004 vaccine group; A, peak Doppler blood inflow velocity across mitral valve during late diastole; CO, cardiac output; Con, the control group; E, peak Doppler blood inflow velocity across mitral valve during early diastole; HFD + L-NAME, the HFD + L-NAME group; HR, heart rate; IVRT, isovolumic relaxation time; LV, left ventricular; LVAWs, left ventricular end-systolic anterior wall; LVAWd, left ventricular end-diastolic anterior wall; LVEDV, left ventricular end-diastolic volume; LVEF, left ventricular ejection fraction; LVESD, left ventricular end-systolic volume; LVFS, left ventricular fractional shortening; LVIDs, left ventricular internal systolic diameter; LVIDd, left ventricular internal diastolic diameter; LVPWs, left ventricular end-systolic posterior wall; LVPWd, left ventricular end-diastolic posterior wall; SV, stroke volume. *P<0.05 and #P<0.01 vs the HFD+L-NAME group.

| **Supplementary Table 2**. Primers sequence for quantitative real-time PCR. | | |
| --- | --- | --- |
| **Molecules** | **Sequence (5’-3’)** | |
| GAPDH | Forward | ACTCTTCCACCTTCGATGCC |
|  | Reverse | TGGGATAGGGCCTCTCTTGC |
| ANP | Forward | GCTTCCAGGCCATATTGGAG |
|  | Reverse | GGGGGCATGACCTCATCTT |
| BNP | Forward | GAGGTCACTCCTATCCTCTGG |
|  | Reverse | GCCATTTCCTCCGACTTTTCTC |
| α1A-AR | Forward | CATCCTGGTTATGTACTGTCGA |
|  | Reverse | GACATTTTTACGGTGGATACGG |
| α1B-AR | Forward | CAATGACGACAAAGAATGTGGG |
|  | Reverse | GATGTAGACTCGGCAGTACATG |
| α1D-AR | Forward | GAAATCCAGGGACACAGAGTAG |
|  | Reverse | CCAGAAGATGACCTTGAAGACG |
| α-MHC | Forward | GCCCAGTACCTCCGAAAGTC |
|  | Reverse | GCCTTAACATACTCCTCCTTGTC |
| β-MHC | Forward | ACTGTCAACACTAAGAGGGTCA |
|  | Reverse | TTGGATGATTTGATCTTCCAGGG |
| LXRα | Forward | ACAGAGCTTCGTCCACAAAAG |
|  | Reverse | GCGTGCTCCCTTGATGACA |
| SREBP1 | Forward | CAAGGCCATCGACTACATCCG |
|  | Reverse | CACCACTTCGGGTTTCATGC |
| Fas | Forward | GCGGGTTCGTGAAACTGATAA |
|  | Reverse | GCAAAATGGGCCTCCTTGAT |
| PPARγ | Forward | GGAAGACCACTCGCATTCCTT |
|  | Reverse | GTAATCAGCAACCATTGGGTCA |
| CD36 | Forward | ATGGGCTGTGATCGGAACTG |
|  | Reverse | GTCTTCCCAATAAGCATGTCTCC |
| VLDLR | Forward | GAGTCTGACTTCGTGTGCAAA |
|  | Reverse | GAACCGTCTTCGCAATCAGGA |
| FATP4 | Forward | AAGGTGAAGAGCATCATAACCCT |
|  | Reverse | TCACGCCTTTCATAACACATTCC |
| F4/80 | Forward | TGACTCACCTTGTGGTCCTAA |
|  | Reverse | CTTCCCAGAATCCAGTCTTTCC |
| TNFα | Forward | CAGGCGGTGCCTATGTCTC |
|  | Reverse | CGATCACCCCGAAGTTCAGTAG |
| MCP1 | Forward | TTGAGGACAGACACAGCAGCC |
|  | Reverse | TCACCAATTCCCCTAGCACC |
| Arg1 | Forward | CTCCAAGCCAAAGTCCTTAGAG |
|  | Reverse | GGAGCTGTCATTAGGGACATCA |
| Acrp30 | Forward | GTTCCCAATGTACCCATTCGC |
|  | Reverse | TGTTGCAGTAGAACTTGCCAG |
| FABP4 | Forward | AAGGTGAAGAGCATCATAACCCT |
|  | Reverse | TCACGCCTTTCATAACACATTCC |
| FATP1 | Forward | CGCTTTCTGCGTATCGTCTG |
|  | Reverse | GATGCACGGGATCGTGTCT |
